# Modern dolomite formation caused by seasonal cycling of oxygenic phototrophs and anoxygenic phototrophs in a hypersaline sabkha

**DOI:** 10.1038/s41598-021-83676-1

**Published:** 2021-02-18

**Authors:** Zach A. Diloreto, Sanchit Garg, Tomaso R. R. Bontognali, Maria Dittrich

**Affiliations:** 1 Department of Physical and Environmental Sciences, Biogeochemistry Group, University of Toronto Scarborough, Toronto, Canada; 2 Space Exploration Institute, Fbg de l’Hopital 68, 2002 Neuchâtel, Switzerland; 3 Department of Environmental Sciences, University of Basel, Klingelbergstrasse 27, Basel, Switzerland

**Keywords:** Carbon cycle, Mineralogy, Geochemistry, Environmental microbiology, Microbial ecology

## Abstract

The “Dolomite Problem” has been a controversy for over a century, owing to massive assemblages of low-temperature dolomite in ancient rocks with little dolomite forming today despite favorable geochemical conditions. Experiments show that microbes and their exopolymeric substances (EPS) nucleate dolomite. However, factors controlling ancient abundances of dolomite can still not be explained. To decode the enigma of ancient dolomite, we examined a modern dolomite forming environment, and found that a cyclic shift in microbial community between cyanobacteria and anoxygenic phototrophs creates EPS suited to dolomite precipitation. Specifically, EPS show an increased concentration of carboxylic functional groups as microbial composition cycles from cyanobacterial to anoxygenic phototroph driven communities at low-and high- salinity, respectively. Comparing these results to other low-T forming environments suggests that large turnover of organic material under anoxic conditions is an important driver of the process. Consequently, the shift in atmospheric oxygen throughout Earth’s history may explain important aspects of “The Dolomite Problem”. Our results provide new context for the interpretation of dolomite throughout Earth’s history.

## Introduction

Low-temperature (low-t) dolomite has been an enigmatic mineral for over a century and the focus of a long-lasting dispute known as a “the dolomite problem”. The puzzle of low-t dolomite stems from its considerable abundance in ancient sedimentary rocks, while its presence inexplicably decreases in younger sequences^1–3^. Findings on synthesis of low-t dolomite have shown that abiotic formation at low temperature is impossible without any heterogeneous surfaces, e.g., seeds^2,4–7^. This includes biological surfaces such as EPS^2,4–6^ and abiotic surfaces such as clays which have been shown to facilitate the formation of proto-dolomite^7^. The lack of precipitation was thought to be caused by inhibitors of reaction kinetics such as cation desolvation^8^, an absence of nucleation sites^9^, inhibition by sulfate ion, and formation of neutral MgSO_4_ over dolomite^4^. The question about kinetic inhibition seemed to be addressed with the observation that low-t dolomite could form in the presence of sulfate-reducing bacteria^10,11^. Later on, multiple laboratory studies demonstrated that low-t dolomite precipitation could occur in presence of microbes under multiple metabolic pathways including aerobic respiration^12^, methanogenesis^13^, methane oxidation^14^, and even sulfide oxidation^15^. More recent findings have suggested that organic material of microbial origin was responsible for catalyzing low-t dolomite precipitation^16–18^. Specifically, low-t Mg-dolomite precipitation under surficial conditions could be catalyzed by the presence of EPS^18–21^. The pathway of this process was later proposed as a possible dehydration of Mg^2+^ facilitated by carboxylic groups which act as a nucleation sites for overcoming kinetic barriers^6,13^. Additionally, laboratory experiments have shown that salinity also plays a role in low-t dolomite precipitation by increasing Mg ions available in addition to increasing the amount of EPS produced and available for nucleation^23–27^.

Although these findings reinforced the view that microbial life, including EPS, represent an important common denominator for the mechanism of low-t dolomite formation, fundamental aspects of this mineralization process throughout Earth’s history remain unclear. In particular, although several environmentally ubiquitous microbial species have been reported to mediate dolomite during laboratory experiments, dolomite formation is not a common process in modern times. Even more intriguing is that while the studied microorganisms represent different metabolic pathways and thus, are present in a wide range of geochemical regimes, dolomite can be found only under very specific environmental conditions. Example of such specific environments include hypersaline settings such as coastal lagoons^10,11^, evaporative lakes^28^, alkaline lakes^29^ and sabkhas^18,19,30,31^, as well as hemipelagic or “Deep-Sea” environments that occur on continental margins^32–34^ and “cold-seeps”^35,36^. Consequently, laboratory culture experiments, although being a generally valuable approach to discover the mechanisms, are not capable of capturing the complexity of natural dolomite-forming environments, in which several microbial metabolisms simultaneously interact with environmental factors changing dynamically (e.g., hourly, diurnal, monthly, seasonal, long-term climatic fluctuations). By integrating these observations under natural conditions light can be shed on these interactions and their influence on the mechanisms of low-t dolomite formation.

Thus, we have conducted an *in-situ* field study of dolomite-forming microbial mats that colonize the intertidal zone of the Khor Al Adaid sabkha in Qatar. This type of coastal sabkha, where low-t dolomite forms in association with other common evaporitic sedimentary facies, is considered as an excellent modern analogue for many ancient, carbonate-rich sedimentary sequences^37,38^. Instead of using a “snap-shot-type” approach based on a single field campaign, the same microbial mats were monitored and sampled in different seasons during a period of three years. This holistic approach provided new insight that, as discussed in the paper, shed new light on the factors that may have controlled the uneven distribution of dolomite throughout the geological record.

## Results

**Figure 1:**
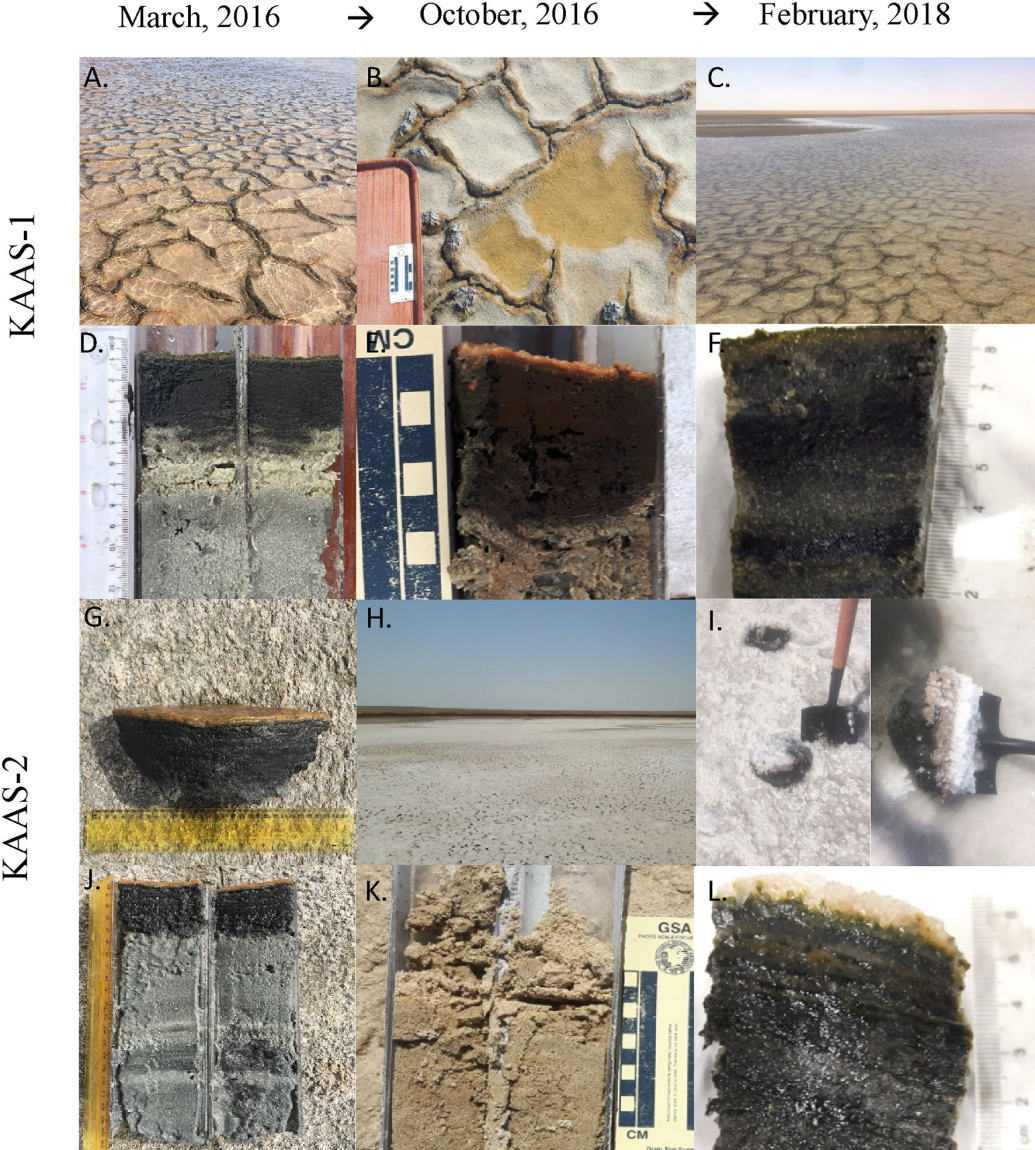
**(A**) Surface of KAAS-1 during March 2016. (**B**) Surface of KAAS-1October 2016. (**C**) Surface of KAAS-1 February 2018. (**D**) Vertical profile of KAAS-1 during March 2016 consisting of the thin upper green layer with intermixed pink and red layers (≈0.5 cm), the thicker middle black layer (≈4.5 cm), and the bottom-most grey layers composing the rest of the profile. (**E**) Vertical profile of KAAS-1 October 2016 with the uppermost orange layer and intermixed pink and orange layer (≈0.5 cm), the middle black layer (≈5 cm), and upper (6 cm), middle (8 cm) and lower grey (> 8 cm) layers. **(F**) Vertical profile of KAAS-1 in February 2018 with green, red and orange layers intermixed (≈0.5 cm), middle black layer (5 cm) and bottom grey layer (> 5 cm) (**G**) Surface of KAAS-2 during March 2016 with extracted portion of mat (middle). (**H**) Dried out surface of KAAS-2 October 2016. (**I**) Salt encrusted surface of KAAS-2 February 2018 and extracted mat portion (left). (**J**) Vertical profile of KAAS-2 during March 2016 showing the orange and red layers (≈0.2 cm), middle black layer (4 cm), and bottom grey layer (> 4 cm). (**K**) Vertical profile of KAAS-2 in October 2016 showing a surficial crust and desiccated core. (**L**) Vertical profile of February 2018 showing a clear-gel underneath the removed salt-encrustation, an intermixed orange, green and red layer (≈1 cm), followed by a middle black layer (3 cm) and the intermixed transition between the black and bottom grey layers (> 3 cm).

The Khor Al-Adaid sabkha is located in the South-Eastern of Qatar. This area has been previously described from an environmental as well as geological perspective^37,38^. Within the sabkha two locations were selected as sampling sites. One within the lower intertidal zone, referred to as KAAS-1, and one within the upper intertidal zone, referred to as KAAS-2. The microbial mats at these two locations were sampled over multiple seasons in March and October of 2016, and February of 2018 (Fig. 1). Some data collected in March of 2016 has been previously presented in^37^.

**Figure 2:**
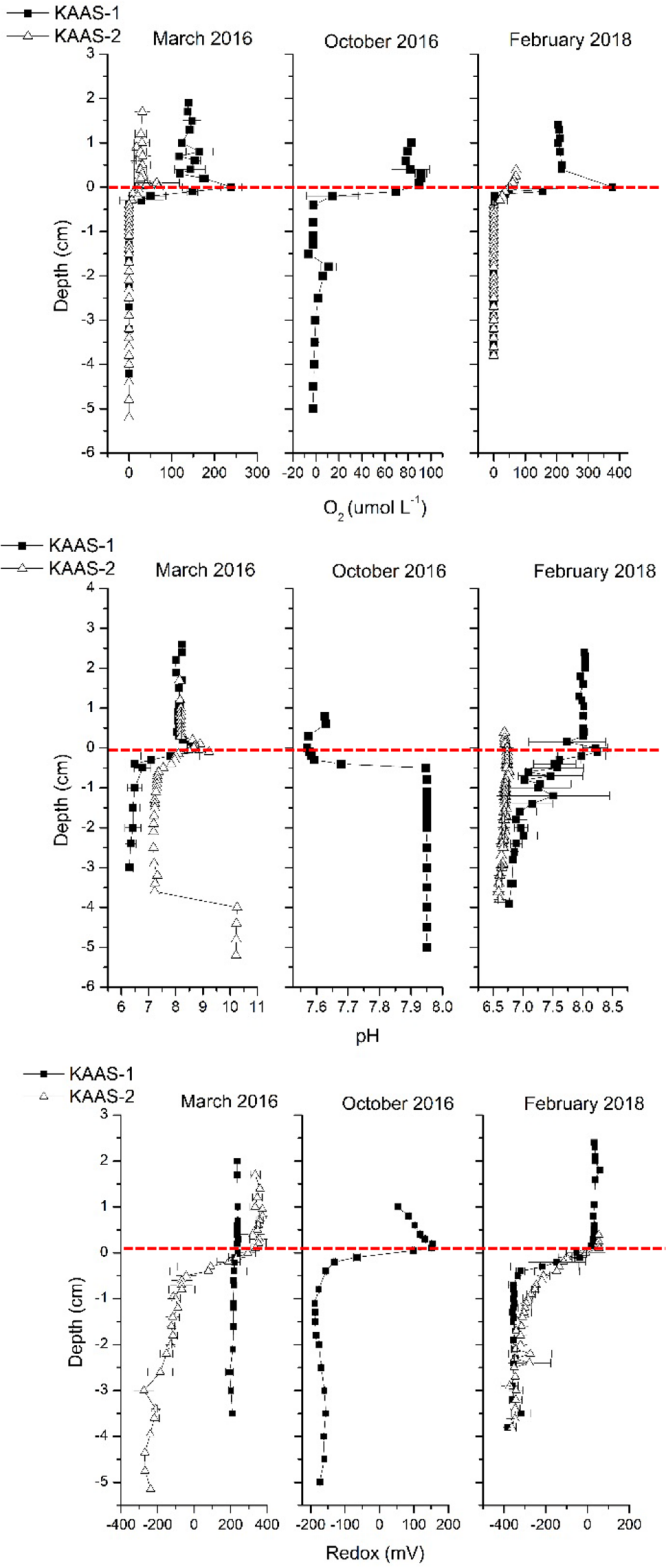
[Top]: Vertical microsensor profiles oxygen [μmol L^−1^] from KAAS-1 and KAAS-2 from March and October 2016, as well as February 2018. [Middle]: Vertical microsensor profiles of pH from KAAS-1 and KAAS-2 from March and October 2016, as well as February 2018. [Bottom]: Vertical microsensor profiles of redox [mV] from KAAS-1 and KAAS-2 from March and October 2016, as well as February 2018.

The geochemistry of water columns and mats at both sites follows a typical progression from photo-oxic to anoxic with depth, oxygen and redox potential follow a typical progression from oxidized to reduced conditions for both mats during all seasons, excluding KAAS-1 in March of 2016 (Fig. 2). pH values in March 2016 and February 2018 are circum-neutral (7.8–8.9) within the water column but show increased pH (8.6–9.2) at the first 5 mm of mat, referred to as the sediment water interface (SWI). Deeper in the mats, in first three centimeters we observed the decreases in pH to values between 6.5 and 7.5. The large increase of pH at depths below 4 cm in KAAS-2 can be linked to a process generating alkalinity or a large accumulation of brine. In our previous study^37^ we reported the presence of halophiles that can only survive at extreme salt concentrations. KAAS-1 during October 2016 shows a different trend with values of 7.6–7.65 in the water column leading up to the SWI interface. Below the SWI within the mat pH values become more basic increasing to 7.95. The trend of pH depths profiles in February 2018 are comparable with those measured in March of 2016.

In October 2016, the mat KAAS-2 has dried out (Fig. 1H,K). Concentrations of major ions as well as salinity, pH and temperature are shown for both sampling sites in (Table S1) and (Table S2). Salinity changes occur in conjunction with temperature increases. In March of 2016 KAAS-1 had consistent salinity values throughout the surface water and mat of ≈ 48‰. In October 2016 salinity of KAAS-1 triples compered to March values within the surface water and almost doubles compered to March values at the SWI and within the mat itself with values of 137.79‰ and 72.57–74.00‰ respectively. February 2018 measurements are similar to values measured in March of 2016, however, salinity in the surface water is much higher, 77.03‰, while at the SWI and within the mat is much lower, 39.46‰ and 25.93‰ respectively. KAAS-2 shows differences in salinity throughout March 2016 and February 2018. March values remain consistent throughout the mat profile ranging from 78.63–81.04‰, while measurements in February are the highest with salinity in the surface waters of 140.12‰, 92.30‰ at the SWI, and 102.76‰ within the mat.

**Figure 3:**
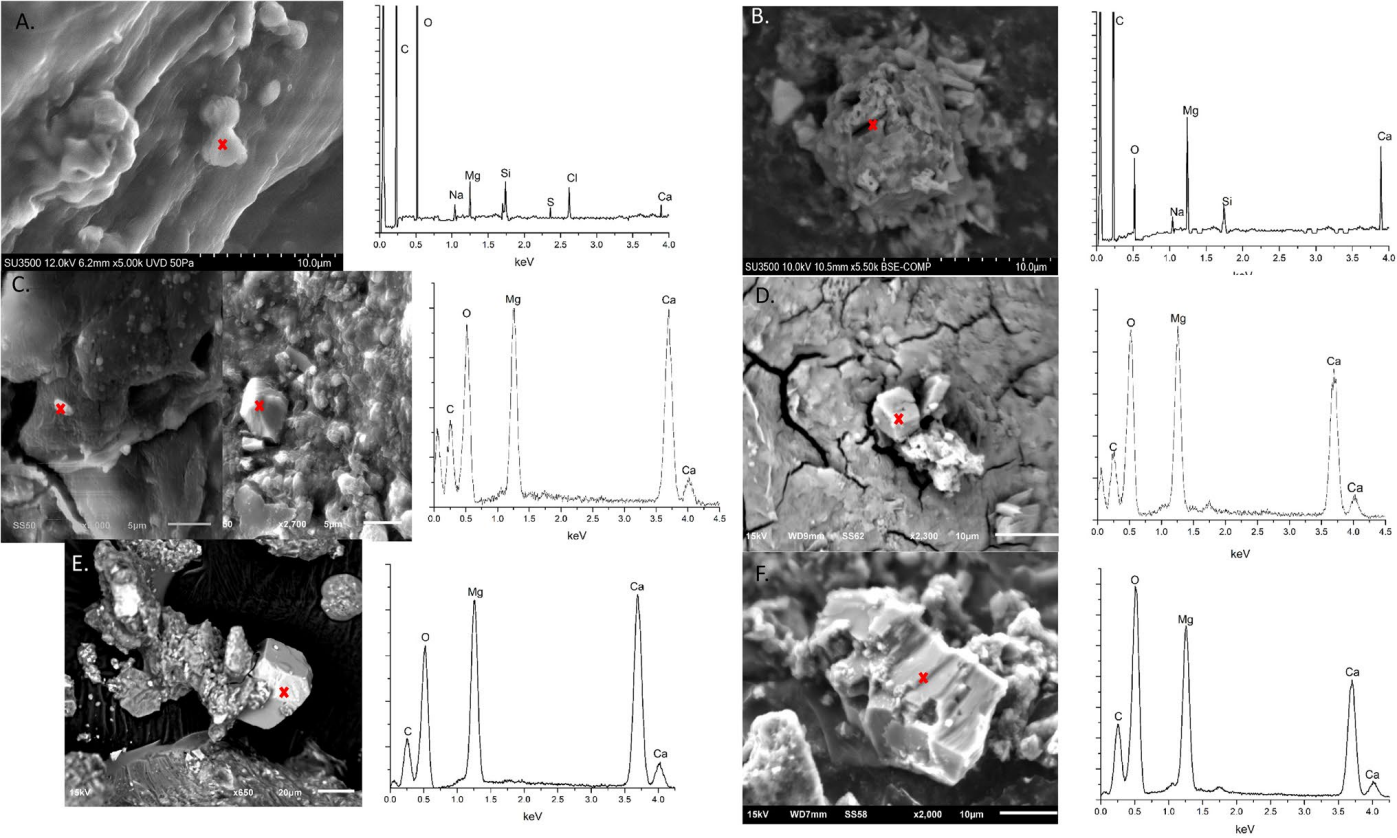
(**A**) Proto-dolomite phase from KAAS-1 during March of 2016 and associated EDS spectra. Area of interest indicated by cross. (**B**) Dolomite phase from KAAS-2 during March of 2016 and associated EDS spectra. Area of interest indicated by cross. (**C**) Proto-dolomite and dolomite phases from KAAS-1 during October of 2016 and associated EDS spectra. Area of interest indicated by cross. (**D**) Dolomite phase from KAAS-2 during October of 2016 and associated EDS spectra. Dolomite only located in the lower layers of the mat at this time. Area of interest indicated by cross. (**E**) Dolomite phase from KAAS-1 during February of 2018 and associated EDS spectra. Area of interest indicated by cross. (**F**) Proto-dolomite phase from KAAS-1 during February of 2018 and associated EDS spectra. Area of interest indicated by cross.

Mineralogy of the mats reveals that dolomite is present within each mat, at every depth, during all sampling season, with the exception of the top layer (or Crust) of KAAS-2 in October 2016. XRD spectra for each season show a distinguish main reflection peak at two theta (2θ) 30.91 which reflects Miller indices hkl = 104 for dolomite as well as ordering peaks at 101 (22.0 for 2θ), 015 (35.19 for 2θ) and, rather less distinguished in some spectra, 021 (43.78 2θ) (Figure S1). The presence of ordering peaks is key in delineating dolomite from disordered dolomite, proto-dolomite, and high Mg calcite^39^. Some of the main reflection peaks exhibit a slight right shift suggesting enrichment in Mg^40^. SEM data displays representative particles with elemental composition similar to dolomite from each of the mats during each season (Fig. 3). The presence of such particles with similar elemental composition to dolomite from KAAS-1 during each season suggests a mix of both proto-dolomite and rhombohedral stoichiometric dolomite and is observed within all layers during each of the measuring seasons. Although it may be present as a precursor mineral, SEM investigations in KAAS-2 revealed no proto-dolomite, instead mostly stacked poly-crystalline aggregates with a composition similar to dolomite, which remain consistent in morphology and crystallinity throughout each sampling season. Large amounts of gypsum and silica (sand from the desert) were detected visually, as well as through XRD and SEM. Generally, more detailed information on these phases was collected during the course of SEM investigations but were not pertinent to this study. There are likely many sulfosalts as well, but confirmation of any specific phases is difficult and speculative due to the high diversity of such phases and complex XRD patterns.

**Figure 4:**
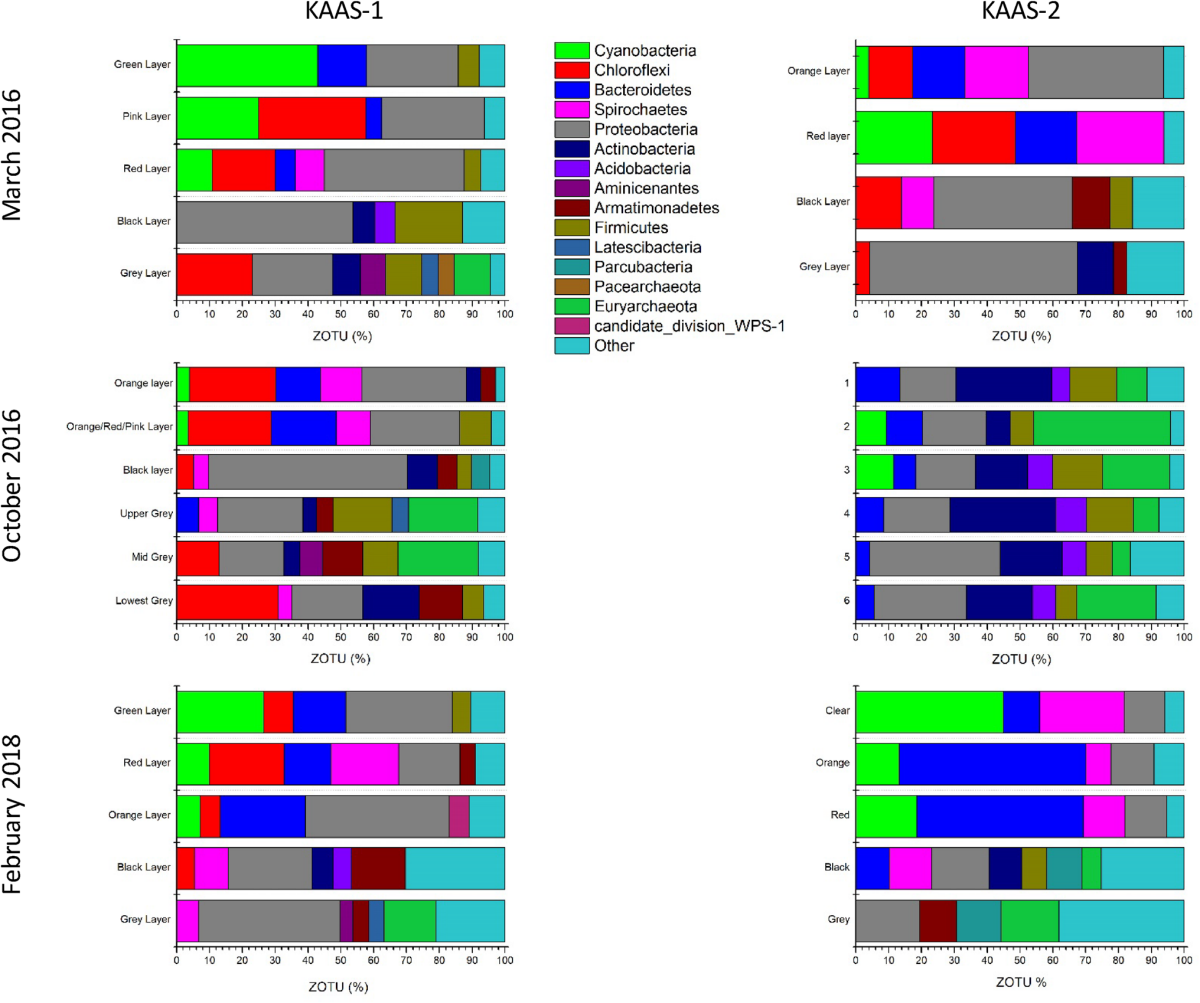
[Top]: Phylum level classification of 16 s rRNA ZOTU abundance (%) from KAAS-1 and KAAS-2 in March of 2016. [Middle]: Phylum level classification of 16 s rRNA ZOTU abundance (%) from KAAS-1 and KAAS-2 in October of 2016. [Bottom]: Phylum level classification of 16 s rRNA ZOTU abundance (%) from KAAS-1 and KAAS-2 in February of 2018.

Microbial community composition at the phylum level for KAAS-1 is shown in (Fig. 4). Community composition at higher and lower taxonomic classifications can be found in (SI ZOTU Files). The most significant changes occur within the uppermost photo-oxic zone of the mats. During March 2016 KAAS-1 mat is denoted by a green uppermost layer which is dominated by cyanobacterial sequences (42.99%) with large abundances of *Proteobacteria* (27.82%), and *Bacteriodetes* (14.92%), and minor amounts of *Firmicutes* (6.36%) within the upper green layer. The composition of the following pink layer shows a significant decrease in cyanobacterial sequences (24.89%), while *Chloroflexi* flourish to 32.7%, accompanied by a minor increase in *Proteobacteria* (31.25%) and decline in *Bacteroidetes* (4.79%). In October 2016 the uppermost layer of KAAS-1 is dominated by *Proteobacteria* (31.86%), *Chloroflexi* (26.38%), with almost equal amount of *Bacteroidetes* (13.55%), and *Spirochaetes* (12.57%). The following mixed red/pink layer shows similar composition with abundances of *Proteobacteria* (27.03%), *Chloroflexi* (25.32%), *Bacteroidetes* (19.79%), and *Spirochaetes* (10.41%). In February 2018 KAAS-1 reverts to a comparable composition as seen in March of 2016. The top layer is mostly a green coloration and its microbial community is dominated by cyanobacterial sequences (26.49%). There are also abundant sequences of *Chloroflexi* (9.04%), *Bacteroidetes* (15.92%), *Proteobacteria* (32.44%), and *Firmicutes* (5.73%). The proceeding red layer denotes a decrease in *Cyanobacteria* sequences (10.01%), and *Proteobacteria* (18.69%), but an increase in *Chloroflexi* (22.78%) similar abundances of *Bacteroidetes* (14.10%) as the previous layer, and the appearance of *Spirochaetes* (20.65%). In all three seasons the microbial community of the lower most layers (red/black/grey) show compositions indicative of reduced conditions with an increased amount of *Proteobacteria* and *Archaea* with depth.

KAAS-2 also undergoes the most pronounced changes in the upper photo-oxic zone of the mats. During March 2016 is denoted by a bright orange uppermost layer which has a microbial community consisting of *Cyanobacteria* (3.9%), *Chloroflexi* (13.51%), *Bacteroidetes* (15.81%), *Spirochaetes* (19.32%), and *Proteobacteria* (41.13%). Below the orange layer is an intermittent red layer consisting of *Cyanobacteria* (23.21%), *Chloroflexi* (25.39%), *Bacteroidetes* (18.62%), and *Spirochaetes* (26.55%). During October 2016 the community composition drastically changes in KAAS-2. Within the uppermost “crust” there are high abundances of *Proteobacteria* (16.29%), *Bacteroidetes* (13.42%), *Actinobacteria* (29.28%), *Frimicutes* (14.18%), and *Euryarchaeota* (9.33%). This composition is similar throughout the entire profile. In February of 2018 KAAS-2 has a clear top layer directly beneath the precipitated salt crystals. The composition of this clear layer is dominated by *Cyanobacteria* (44.92%), *Bacteroidetes* (11.00%), *Spriochaetes* (25.86%), and *Proteobacteria* (12.29%). Underneath this is a layer with an intermixing of green, pink and orange. Within this layer the microbial community is composed of *Cyanobacteria* (13.19%), *Bacteroidetes* (56.77%), *Spirochaetes* (7.72%), and *Proteobacteria* (13.08%). In conjunction with this top layer is an intermittent red layer with similar composition, consisting of *Cyanobacteria* (18.58%), *Bacteroidetes* (50.68%), *Spriochaetes (*12.60%), and *Proteobacteria* (12.79%).

In all of the sampling seasons the amount of proteins that comprise purifiable EPS are low in comparison to carbohydrates and uronic acids often falling near the detection limit of the employed Bradford assay (1ug/g for micro-assay)^41^. Uronic acid concentration is markedly higher during times of increased salinity, and a majority of all three fractions appear concentrated in the uppermost portion of the mat. Total concentrations of each functional group are summarized in (Table 1). The spectra analysis suggests that there are four assemblages of functional groups based on pKa ranges carboxylic (3–5.8), phosphoryl (6–8), amine (8–9) and hydroxyl (9–10)^42–45^ (Figure S4).

**Table 1:** Summary of Total functional group concentrations over their pKa ranges from the uppermost mat layers during each sampling season. These measurements originate from EPS extracted from the green and orange layers or top 0.5 cm of each mat given a particular sampling season.

Functional Group	pKa Range	KAAS-1 March 2016	KAAS-2 March 2016	KAAS-1 October 2016	KAAS-1 February 2018	KAAS-2 February 2018
		Average L_T_ (mMol/g EPS)	Average L_T_ (mMol/g EPS)	Average L_T_ (mMol/g EPS)	Average L_T_ (mMol/g EPS)	Average L_T_ (mMol/g EPS)
Carboxyl	3–5.8	0.113 ± 0.08	0.173 ± 0.04	0.461 ± 0.23	0.075 ± 0.03	0.091 ± 0.05
Phosphoryl	6–8	0.149 ± 0.01	0.103 ± 0.04	0.260 ± 0.32	0.044 ± 0.02	0.020 ± 0.01
Amine	8–9	0.845 ± 0.25	0.224 ± 0.03	0.721 ± 0.48	0.545 ± 0.01	0.223 ± 0.02
Amine (hydroxyl)	9–10					

In March of 2016 EPS extracted from KAAS-1 contains 0.113 ± 0.08 mMol g^−1^ carboxylic functional groups, 0.149 ± 0.01 mMol g^−1^ phosphoryl functional groups, and 0.845 ± 0.25 mMol g^−1^ amine functional groups. EPS extracted from KAAS-1 during October of 2016 exhibit much more variability in composition and mark an increase in carboxylic groups up to 0.461 ± 0.23 mMol g^−1^, in phosphoryl groups up to 0.260 ± 0.32 mMol g^−1^, and a decrease to 0.721 ± 0.48 mMol g^−1^ in amine groups. In February of 2018 functional group density experiences an overall decrease with concentrations of 0.075 ± 0.03 mMol g^−1^ for carboxylic groups, 0.044 ± 0.02 mMol g^−1^ for phosphoryl groups, and 0.545 ± 0.01 mMol g^−1^ for amine groups. Similar to KAAS-1 the amount of extractable and purifiable proteins from KAAS-2 during all sampling seasons is negligible with some values below the assay detection limit. KAAS-2 shows increased concentrations of the uronic acid fraction during times of high salinity, this trend we also observed in KAAS-1. EPS extracted from KAAS-2 in March of 2016 has concentrations of 0.173 ± 0.04 mMol g^−1^ for carboxylic groups, 0.103 ± 0.04 mMol g^−1^ for phosphoryl groups, and 0.224 ± 0.03 mMol g^−1^ for amine groups. In October of 2016 there was no extractable EPS from the dried-out mat. During February 2018 EPS sampled from KAAS-2 had functional group concentrations of 0.091 ± 0.05 mMol g^−1^ for carboxylic groups, 0.020 ± 0.01 mMol g^−1^ for phosphoryl groups, and 0.223 ± 0.02 mMol g^−1^ for amine groups. Quality control of titration data for KAAS-1 and KAAS-2 show good agreement between both measured and modeled titrations (Figure S3).

## Discussion

The Khor Al-Adaid sabkha undergoes distinct seasonal fluctuations as a result of the local climate^37^. Air temperature changes of 5–10 °C are the root cause of the differences between mats. There are a number of smaller scale interlinked geographical factors that can influence the mat environment such as water recharge rate and source, topography, and tidal range; however, the effect of these factors is probably limited. Instead, a more appropriate frame of reference is the geomorphology of the sampling sites, or more specifically the intertidal nature of the environment. The position in intertidal zonation strongly governs mat morphology, evaporative effects, and water exchange^46,47^. Our previous study^37^ reveals that KAAS-1 falls within the lower intertidal zone, while KAAS-2 is located within the mid to upper intertidal zone, subjecting KAAS-2 to more extreme changes in environmental conditions.

Among all environmental factors, our observations show that salinity, which fluctuates drastically between seasons, appears to be a major driver in low-t dolomite precipitation. Salinity influences geochemical parameters by governing solution chemistry and consequently, saturation indices of the mineralogical phases^48^. Conditions of supersaturation with respect to dolomite are, however, insufficient for dolomite formation, which is kinetically inhibited at low temperatures^2^. In light of the proposed role of microbial produced EPS in low-t dolomite formation, it is critical to evaluate the role salinity fluctuations in the expression of the microbial community and consequences for EPS characteristics.

Our observations in sabkha environment show that the mat microbial communities respond strongly to the fluctuations in salinity, undergoing significant changes during lower and higher saline conditions. The changes to microbial composition are most pronounced within SWI zone in the uppermost layers of both microbial mats. We observed that during cool periods microbial mats at KAAS-1 are dominated by *Cyanobacteria* (oxygenic phototrophs) within its uppermost layers, while microbial mats at KAAS-2 are dominated by *Chloroflexi* (anoxygenic phototrophs). Interestingly, anoxygenic phototrophs occur where oxygen is still saturated. Under such conditions, anoxygenic phototrophy is suppressed, which most probable cause the organisms to respire heterotrophically^49^. During warm periods KAAS-1 experiences salinity resembling KAAS-2 during cool periods, which is reflected by a comparable microbial community composition. Once the temperature becomes cooler again, the microbial mat composition in KAAS-1 returns similar to that found in March, while KAAS-2 remains dominated by heterotrophic organisms (*Salinibacter*, e.g., 50) reflecting an ecological succession or hysteresis effect. The cyclic nature of the microbial community composition suggests a reproducible sequence of events that operate over longer time periods, especially when considering the re-establishment of KAAS-2 after complete desiccation.

This hysteresis effect in microbial communities is accompanied by cyclic changes in EPS composition. The concurrent molecular characterization of EPS and analysis of microbial compositions during three seasons reveal that microbial mat dynamics can be closely linked to the functional groups of EPS, their densities as well as the amount of EPS. We found that high salinity, and the shift from *Cyanobacteria* to *Chloroflexi,* or *Salinibacter* was coexisting with a rise in the amount of extractable EPS, as well as an increase in the fraction carboxylic functional groups, the proposed ligands for the formation of low-t dolomite^6,13^. Cumulatively, these results indicate that by shifting a microbial community from oxygenic phototrophs to heterotrophs as a result of salinity creates EPS favorable to dolomite nucleation.

The previous studies which focused either on microbial community analysis in the Arabian Peninsula, Pacific atolls, Mexico, Spain, Washington (USA)^51–56,58,61^ or geochemical conditions in Spain and Cuba^57,59,60^, documented similar environmental interactions between microbial communities succession and salinity in microbial mats. This fact suggests that our observations in the Khor Al-Adiad sabkhas are not locally restricted. However, the lack of multi-seasonal and interdisciplinary approach, as well as limited attention to mineral-microbe interactions hinder the previous studies to draw the conclusions about the biogeochemical dynamics in microbial composition, consequences for EPS quantity and quality and at long last on low-t dolomite formation.

The discovery of this truly biogeochemical interplay between bio- and geosphere in our field studies is even more astonishing, since many investigators observed this particular pathway for low-t dolomite formation during a transition in microbial mats driven by environmental factors, especially salinity^57–60^. For example, very recent in the playa lakes, maximum appearance of dolomite was reported to occur concurrently with dominance of anoxygenic phototrophs^57,58^. In another study, increased carbonate mineralization was documented as microbial mats experienced a transition from oxic to anoxic conditions^59,60^. These types of dynamic fluctuations do not appear to be unique to surficial examples of low-T dolomite forming environments. Indeed, literature examining the occurrence of hemipelagic or “deep-sea” dolomite describes low-T dolomite formation in environments rich in organic material in the presence anaerobic heterotrophic organisms.

Hemipelagic or “deep-sea” low-t dolomite has been documented in a variety of locales, generally in areas of upwelling across the globe, including, for example: the west coast of Africa^32^, the west coast of south America in Peru^33,34^, and cold-seeps in the Gulf of Mexico^35,36^. Due to the nature of these sites, samples on which these studies are based consist of drill cores and microbial activity is mostly inferred through isotopic analysis of carbonates and pore waters, such as δ^13^C, δ^18^O, carbonate-associated sulfate (CAS), or stable magnesium isotopes including δ^25^Mg and δ^26^Mg. Instead, data based on direct measurements of the microbial community, which would provide consistent and valuable information into the occurrences of low-T dolomite under these conditions, remains limited. It has been observed that strong depletion in 13C values may suggest biogenic methane as a main carbon source^35^. Similar 13C depletion results as well as δ^13^C and CAS ratios were also obtained from dolomite bearing samples^36^. It was suggested that dolomite was formed in the shallow subseafloor, due to sulfate-driven anaerobic methane oxidation (AOM), which is performed by a consortium of sulfate-reducing bacteria^36,77^. The combination of these metabolism results in the depletion of sulfate and buildup of sulfide in a zone referred to as the deep sulphate-methane transition zone (SMTZ). It was noted that the kinetic barrier being overcome under these conditions was still unclear^36^. It is possible that the high turnover of organic material by AOM prokaryotes results in a similar effect as the transition between oxygenic and anoxygenic organism observed in the sabkha environment. Unfortunately, without a direct characterization of organic material from the seep environment, this explanation remains hypothetical.

A closer examination of the process of AOM (1) shows the processes can shift equilibria toward carbonate formation.

1 $$ {\text{CH}}_{4} + {\text{SO}}_{4}^{2 - } \to {\text{HCO}}_{3}^{ - } + {\text{HS}}^{ - } + {\text{H}}_{2} {\text{O}} $$

It has been suggested that the increase in alkalinity and HCO_3_ result in more CO_3_^2−^ to compensate for the increased CO_2_ created^32^. Although this is a sound argument, how the precipitation process overcomes the kinetic barriers preventing mineral formation remains unknown. Data from the sabkha study provides a possible explanation as it sheds light on the biogeochemical interplay between a synchronized transition in the geochemical conditions, microbial composition, EPS (or, more generally, organic material) and its role for low-t dolomite formation. In the case of hemipelagic low-T dolomite, the presence of large abundances of additional abiotic nucleation surfaces, such as clays^7^, could further facilitate low-T dolomite precipitation. However, until now a direct examination of naturally occurring organic material and its influence on low-t dolomite formation have not been systematically investigated.

Referencing the sabkha microbial mats, a phase of dominance of *Cyanobacteria* can be described as a growing microbial mat (GMM) in opposite to a period of time of decaying microbial mat (DMM) with high abundances of anoxygenic phototrophs and heterotrophs [e.g.,^57^]. The cyclic transitions between these two phases of GMM and DMM are linked to changes in salinity, a community shift to heterotrophic metabolisms, synchronized with molecular changes in functional groups of EPS and, finally, with more crystalline morphologies of dolomite-like phases. Within the sabkha’s microbial mats of our study, such solid phases evolve from proto-dolomite or stacked aggregates to fully formed rhombs. It has been hypothesized that rising salinity or the transition from oxic to anoxic conditions cause EPS degradation by heterotrophic activity resulting in the release of Ca and Mg into solution and freeing of previously bound functional groups responsible for carbonates nucleation^47–60,62^ In the case of hemipelagic low-t dolomite, this process is also characterized by an increase in alkalinity associated with AOM. Additionally, it should be noted that, within the Khor-Al Adaid sabkhas, massive, continuous layers of low-T dolomite have not been observed but are present within the northern Dohat Faishakh sabkhas^31^ and sabkhas of Abu Dhabi^18^. These sites along with many other low-T dolomite forming environments^10,11,28–35^ show continuous layers of dolomite 10 s of centimeters thick formed under what can be considered similar conditions. The sabkha environments in Abu Dhabi and northern Qatar [e.g.,^18,31^] are older, more extensive and result in thicker profiles (≈40 cm) than the Khor Al-Adaid sabkha. Additionally, much of the dolomite found in these environments is at lower depths (10–40 cm) and has been proposed to initially form at the surface as proto-dolomite and disordered dolomite, the same as the Khor Al-Adaid mat, then progress and accumulate to dolomite due to burial and diagenetic “ageing”. The observed lack of large continuous layers of low-T dolomite in this study could be due to sample variability although is more likely due to discontinuous presence of active microbial mats (for example the completely desiccated KAAS-2 mat during the October 2016 sampling), which would result in less accumulation and decreasing burial rates. Despite the lack of continuous, thick layers of low-T dolomite, the dolomite-like phases formed within these mats is authigenic^37^. Observations from the sabkha highlight the mechanism of initial nucleation and indicate a more nuanced process of low-T dolomite formation than previously proposed.

We observed that the high abundance of anoxygenic phototrophs or *Bacteroidetes*, *Chloroflexi* and *Salinibacter*, occur in zones saturated with oxygen. Under such conditions, anoxygenic phototrophy is suppressed, which may cause *Chloroflexi* to respire heterotrophically^49^, creating a comparable metabolic dynamic. Ca and Mg and an augmented alkalinity can surely cause an additional increase in dolomite saturation index. But, this is not sufficient for dolomite precipitation, which is kinetically inhibited^29^. The functional groups of EPS may support overcoming the kinetic barriers that prevent Mg to be incorporated into the carbonate mineral at low temperature. Once this kinetic barrier has been overcome the formation of proto-dolomite and dolomite would proceed in the upper layers of the sabkha through an adsorption-displacement mechanism onto growing Ca-Mg carbonate^3,7^ followed by a currently poorly defined “ageing” process to form ordered dolomite [e.g.,^10^]. The ageing process is a combination of diagenesis and time, which would proceed at lower depths in the mat as it accumulates. Overall, we suggest that the presence of EPS with a specific composition, rather than cation release during EPS degradation or alkalinity increases [e.g.,^29^] during AOM, is the key factor for the nucleation of dolomite during environmental transitions, like the described SMTZ or GMM and DMM.

**Figure 5:**
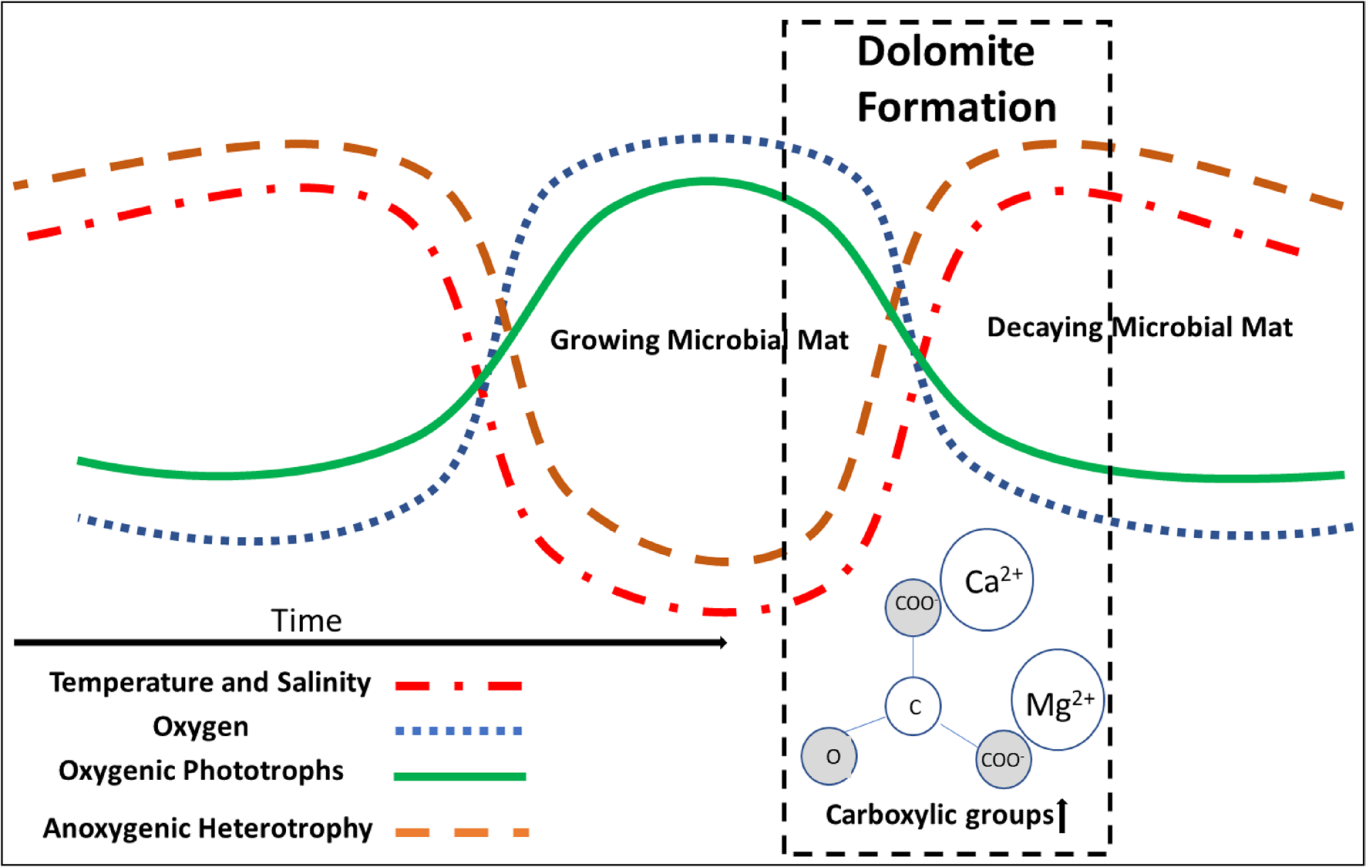
Conceptual diagram of proposed multi-seasonal low-t dolomite formation. Shifting environmental conditions cycle between higher and lower temperature and salinity. This results in the cycling of a growing and decaying microbial mat with low-t dolomite formation facilitated during the transition between growing and decaying conditions.

Our findings indicate that geochemical driven cycles of growth and decay within specific microbial communities such as oxygenic and anoxygenic phototrophs or heterotrophs, promote low-t dolomite formation. In other words, biogeochemical conditions coupled with a specific type of microbial community creates EPS that has a higher and lower affinity for low-t dolomite formation (Fig. 5). This hypothesis is in agreement with both sites for low-t dolomite precipitation, as well as current microbial models, which attribute low-t dolomite precipitation to low oxygen concentrations and the activity of sulfate-reducing bacteria^10,63–65^. These models suggest that low-t dolomite precipitation proceeds as the kinetic barriers caused by SO_4_^2−^ are removed. However, experiments have shown that SO_4_^2−^ presents less of a problem than previously thought^66,67^. Instead, we propose that in environments with low oxygen and high organic matter turnover, heterotrophic microbes become more pronounced and consume organic material creating substrates with compositions better suited to dolomite precipitation. This is corroborated by recent laboratory studies that have suggested a similar “new-way” of producing low-t dolomite in the presence of anoxygenic strains^26,68^, which is also in agreement with our concept of dolomite formation. Our hypothesis is soundly corroborated in modern environments and laboratory experiments, but attempting to apply this model to paleo-studies regarding low-t dolomite can allow us to evaluate its applicability over longer time scales.

From a geological perspective, a fundamental consequence of our concept is that the interplay between production and consumption of EPS in conjunction with the GMM to DMM transition is an important factor in low-T dolomite formation. Consonant with our hypothesis, are ancient dolomites that have been described in several recent studies. For example, formation of massive Precambrian carbonate platforms is suggested to be connected to the interaction between cyanobacteria and heterotrophic bacteria or anoxygenic bacteria metabolizing heterotrophically (heterotrophic sulfate reduction) by production of H_2_O and CO_3_^2−^ caused by dissociation of ammonia and hydroxyl ions during buffering and may have also raised carbonate alkalinity^69^. Additionally, a recently compiled dataset of ocean chemistry and carbonate strata from the late Archaean through the Cambrian, shows similar links between the distribution of aerobic and anaerobic metabolisms and dolomite^70^. This study reported higher abundances of dolomite at all ocean depths during the Archean, but a higher affinity for dolomite in shallow sedimentary environments as time progressed. This occurred in conjunction with the spread of aerobic organisms across the seafloor which may have restricted anaerobic microorganisms to shallow areas^70^. Given that the change in ocean geochemistry and redox conditions, as well as microbial metabolism, these secular changes may have resulted in large turnovers of organic material, a process analogous to the seasonal fluctuations observed within the Khor-Al Adaid mats. Dolomite formation in the quaternary has also been linked to changes in water and redox chemistry that led to elevated cell mortality and organic matter turnover^29^. Thus, it seems that the interactions between microbial mats impacted by changing redox conditions in the environment and consequent microbial community are imperative for dolomite formation through geological time. Specifically, our observations in the sabkhas as well as paleoenvironmental and laboratory studies have indicated that occurrence anoxygenic heterotrophs may play a key role in low-T dolomite formation aside from sulfate-reduction^15,23,26,27,29,32–36,66–68^.

More specific paleo-environmental also corroborate our findings and the compilation of existing data discovers striking similarities between the modern environments and ancient low-t dolomite locations. The sedimentological studies on ancient dolomite, namely cap dolostones approximately 635 Ma old, from the Puga Formation in Brazil, confirm our concept even for dolomite formation over a much larger extent. A progressive change in temperature and salinity has been isotopically identified in cap dolostones from the Puga Formation in Brazil^71^. Within these dolostone caps a stromatolitic environment dominated by cyanobacteria existed and caps may have been deposited as conditions transitioned from anoxic to suboxic as suggested by the observed sedimentary features^71^. Another very recent study on the same post-glacial dolostone cap rocks discovered enrichment of newly identified biomarker 25,28-bisnorgammacerane (25,28-BNG) which can be formed as a result of a transition of microbial mat dominated by cyanobacteria to one dominated by heterotrophs^72^. Further paleo-evidence for the proposed mechanism facilitating low-t dolomite formation comes from studies that examined dolocretes were formed within the Hammersley Basin during the quaternary age (37 to > 45 ka) and Late Miocene and Pliocene (5.3–3.5 ma)^73,74^. In both cases, dolocrete formation is suggested to be stimulated by more saline evaporitic paleoenvironmental conditions, possibly playa lakes or saline mudflats, as evidenced by δ^18^O values. Interestingly, the most recent study observed extensive fossilized EPS and organic filaments in more shallow sections of the same cores^74^ indicating a possible presence of microbial mats at the dolomite forming locations, even though no information about microbial composition exists. The paleoenvironment in which this occurred looks remarkably resembles the Khor Al-Adaid sabkha. Consequently, it is probable that the higher saline conditions would have promoted similar dynamics in which a growing mat would have undergone degradation stimulating dolomite precipitation.

Overall, our observations demonstrate that environmental fluctuations, in particular salinity, and an accompanying transition from oxygenic to anoxygenic heterotrophic dominance in microbial mats created an organic substrate (EPS) particularly suited to facilitate low-t dolomite precipitation. Specifically, increased salinity leads to a community dominated by anoxygenic phototrophs which degrade EPS produced by *cyanobacteria*. This resulted in elevated concentrations of carboxylic functional groups, which are known to promote Mg dehydration and subsequent incorporation into carbonate minerals. Importantly, this study reveals the interplay between oxygenic and anoxygenic phototrophs and the GMM to DMM is cyclical and may propagate over longer time scales. Currently established models of dolomite precipitation, as well as several modern, and paleoenvironmental studies purport findings that are in agreement with our interpretation of low-t dolomite precipitation and we suggest that low-t dolomite may have decreased with time due to secular changes in the Earths’ atmosphere and ocean chemistry.

## Methods

### Depth profiles of O_2_, pH, redox potential, and salinity

Depth profiles of geochemical characteristics, including O_2_, pH and redox potential, were collected using needle-tip microsensors (Unisense, Denmark) connected to a Unisense 4 channel multimeter. These profiles were measured *in-situ* between 11-2p.m (AST), recorded water temperature was of 30-35ºC. Salinity of surface water at each site was measured using a Metrohm 914 pH/conductometer and conductivity probe with built in temperature sensor (Metrohm part #6.0917.080). Measurements were then converted to salinity using a temperature-corrected calibration curve. Detailed information on sensor configuration and calibration can be found in supplementary data.

### Porewater analysis

Porewater was collected using Rhizons during March and October 2016 (Rhizosphere research products, Netherlands) from pooled water above the mat, as well as using short cores with pre-drilled and covered holes. Porewater from 2018 was collected using prefabricated diffusive gradient thin films or DGT samplers (DGT Research Products, Lancashire, UK). Porewater samples were preserved for Inductively Couple Plasma – Optical Emission Spectrometry (ICP-OES) in 5 ml centrifuge tubes with 5% HNO_3_ from Rhizons, while DGT samples were sealed and both stored at 4 °C until analysis. Detailed information on preparation of pore-water and analysis using ICP-OES can be found in the supplementary data.

### Scanning electron microscopy (SEM), energy-dispersive X-ray spectroscopy (EDS), and X-ray diffraction (XRD) analysis

Morphological examination, elemental composition of samples was performed through a combination of scanning electron microscopy (SEM), and energy-dispersive X-ray spectroscopy (EDS) respectively. The mineralogical characterization was performed by X-ray diffraction (XRD). Details on the specific machines and sample preparation and analysis can be found in the supplementary data.

### 16s rRNA amplicon library preparation and data analysis

Samples of up to 10 g were isolated from short cores for metagenomic analysis following the same procedure from^37^. Cores were sliced under an N_2_ atmosphere using aseptic techniques and preserved via freezing immediately at − 80 °C. Samples were stored at − 80 °C until DNA extraction was performed using a MoBiopower biofilm DNA isolation kit as per the manufacturer’s instructions. DNA isolates were stored at − 20 °C until sequenced. 16S rRNA amplicon sequencing targeted the V4 variable region using the combined bacterial and archaeal primer set of 341F (CCTACGGGNGGCWGCAG) and 785R (GACTACHVGGGTATCTAATCC). Further details on amplicon library preparation and bioinformatic analysis can be found in the supplementary data.

### Extraction and characterization of exopolymeric substances (EPS) from microbial mats

EPS composition throughout each sampling season was evaluated from the uppermost, middle, and bottom layers of each mat (Fig. S2). EPS were extracted and characterized following modified protocols outlined in^75^. Details on extraction procedure and colorimetric analysis of fractions as well as quality control can be found in the supplementary data.

### Potentiometric titrations

Potentiometric titrations were performed to determine acidity constants (Pka) and concentration of functional groups in EPS from microbial mats. All potentiometric titrations were performed on a 905 Titrando auto-titration system (Metrohm) on dynamic endpoint titration (DET) mode in gas-tight-vessel (Metrohm1-50 ml with thermostat jacket). A mass of 20–80 mg of purified EPS were used for each titration with 5 ml of 0.01 M KCl as an electrolyte solution and 0.1 M HCl and 0.01 M NaOH as titrants. Each sample was degassed for 1 h under N_2_ gas prior to titration and was analyzed in triplicate. Following the titration the data was expressed in the form of charge excess which is calculated using the first half following equation^42,76^:

$${Cb}_{j} - {Ca}_{j}+ {\left[{H}^{+}\right]}_{j} - {\left[{OH}^{-}\right]}_{j }= {\sum }_{i=1}^{n}\left(\frac{{K}_{ai}{L}_{Ti}}{{K}_{ai} + {\left[{H}^{+}\right]}_{j}}\right) + S$$

where Ca_j_ and Cb_j_ are the concentrations of acid and base at each j addition of titrant [H^+^]_j_ and [OH^−^]_j_ are the proton concentrations at each j addition of titrant.

The second half of the equation represents the surface binding sites based on charge excess and are determined as a sum of the number (n) of monoprotic ligands (L_Ti_) with resulting in their total concentration along with acidity constants, as well as the constant (S) acid neutralization capacity of the EPS surface. The functional group determination by this method was performed using a linear programming method (LPM)^42,76^ over a pH range of 4–10 at 0.2 unit intervals. Prior to LPM charge excess obtained from titrations was plotted to determine agreement between forward and reverse runs as well as sample replicates.

## Supplementary Information

Supplementary Information 1.

Supplementary Information 2.

Supplementary Information 3.

Supplementary Information 4.

Supplementary Information 5.

## Data Availability

All datasets generated or analyzed will be made available by request in a timely manner to any qualified researcher. 16S rRNA sequences were submitted for individual samples to the NCBI SRA database and will be available under accession numbers PRJNA489272 and PRJNA634701 upon publication.
