# Supplementary material for: Modern dolomite formation caused by seasonal cycling of oxygenic phototrophs and anoxygenic phototrophs in a hypersaline sabkha
Source: Sci Rep. 2021 Feb 18;11:4170. doi: 10.1038/s41598-021-83676-1 (PMC7893050; doi:10.1038/s41598-021-83676-1)
Supplement: Supplementary file 1 — Supplementary Information 1. [file 41598_2021_83676_MOESM1_ESM.docx]

**Supplementary Data for: “Modern dolomite formation caused by seasonal cycling of oxygenic phototrophs and anoxygenic phototrophs in a hypersaline sabkha”**

Zach A. DiLoreto^1*^, Sanchit Garg^1^, Tomaso R.R. Bontognali^2,3^, Maria Dittrich^1^

^1^University of Toronto Scarborough, Department of Physical and Environmental Sciences, Biogeochemistry Group, Toronto, Canada

^2^Space Exploration Institute, Fbg de l’Hopital 68, 2002, Neuchâtel, Switzerland

^3^Department of Environmental Sciences, University of Basel, Klingelbergstrasse 27, Basel, Switzerland

* Zach A Diloreto

**Email:** [zach.diloreto@utoronto.ca](mailto:zach.diloreto@utoronto.ca)

[tomaso.bontognali@space-x.ch](mailto:tomaso.bontognali@space-x.ch)

[sanchit.garg@mail.utoronto.ca](mailto:sanchit.garg@mail.utoronto.ca)

[m.dittrich@utoronto.ca](mailto:m.dittrich@utoronto.ca)

https://orcid.org/0000-0002-0295-2764

# Supplementary Methods

# Depth Profiles of O2, pH, redox potential and salinity

All sensors were calibrated as per the manufacturer’s instructions before measurements were taken (1). O_2_ saturation measurements were calibrated against model calculations (2) for hypersaline conditions. The *in-situ* measurements fall within the values in the calibrated ranges, with the exception of the surface of the mats, which are oversaturated in respect of oxygen; however, this is a common phenomenon due to photosynthetic activity (3). Salinity of surface water at each site was measured using a Metrohm 914 pH/conductometer and conductivity probe with built in temperature sensor (Metrohm part #6.0917.080)). Measurements were then converted to salinity using a temperature-corrected calibration curve. Salinity at depth was also determined via total concentration of ions extracted from porewater in g L^-1^.

# Pore water analysis

Prior to analysis DGT samples had their filter layer, and gel layer removed to expose the binding resin. This resin was cut horizontally into 1mm-10mm strips depending on vertical depth within the mat. 1mm intervals were used from 0-1cm depth, 0.5mm for 1-11cm depths and 1cm for 11-15cm depths. Metals were liberated from DGT by immersing the resin gel in 1M HNO_3_ for 24 hours.

Due to the hypersaline nature of the sampling environment, both pore-water collected by Rhizons and extracted DGT samples were required serial dilutions in HNO_3_. Elemental concentrations for porewater collected in March and October 2016were analysed by from ICP-OES were obtained using an Agilent 720-ES system (Agilent, Canada). Samples for February 2018were analyzed with an Agilent 8800 Triple Quadrupole ICP-MS by using collision and reaction gases to reduce polyatomic interferences (4). External calibration was made with a multi-element mixture prepared in 2% nitric acid. Internal standards were continuously fed inline with the samples and used to account for instrumental drift. The instrument was run in high matrix mode using argon (Ar) gas to internally dilute samples after injection. Elemental concentrations were then back-calculated following the manufacturer’s instructions.

# Scanning Electron Microscopy (SEM), energy-dispersive X-ray spectroscopy (EDS), and X-ray diffraction (XRD) analysis

Samples for SEM analysis were isolated from the distinct layers of each mat **[Figure 1 B, C]** and prepared via a light rinse in distilled water and desiccation for 24 hours prior to analysis. Dolomite isolates from the same depth were also examined (1). Mat samples were examined under variable pressure (50-70 Pa) at 10 kV on a Hitachi SU-3500 SEM (HITACHI, Japan). EDS spectra were collected from areas of interest using an Oxford Instruments X-Max system and AZtec software package. Dolomite samples were examined under the same conditions. XRD was collected using a Phillips XRD system with the following conditions. Cu K-α X-ray source with a wavelength of 1.5406 Å, scanning steps size of 0.02° and angle range of 15.02 to 59.98.

# 16s rRNA amplicon library preparation and data analysis

PCR was performed using the HotStarTaq Plus Master Mix Kit (Qiagen, USA) under the following conditions: 94°C for 3 minutes, followed by 28 cycles of 94°C for 30 seconds, 53°C for 40 seconds, and 72°C for 1 minute, followed by a final elongation step at 72°C for 5 minutes. Post-amplification PCR products were checked for quality in 2 % agarose gel, pooled and then purified using Ampure XP beads. Amplicon sequencing was performed by Mr. DNA (www.mrdnalab.com, Shallowater, TX, USA) using a MiSeq (Illumina) as per the manufacturer’s guidelines. Sequences provided were analysed using a combination of QIIME (5) and Usearch version 10.0 (6). Analysed sequences were joined and demultiplexed in QIIME due to file size. Primers were stripped and sequences less than 200 base pairs were removed using Usearch. Sequences were also filtered and dereplicated using Usearch. To assign operational taxonomic units (OTUs) dereplicated sequences were denoised using the UNOISE algorithm (7) and clustered at 100 % similarity creating zero distance operational taxonomic units (ZOTUs) (8). Taxonomy of assigned ZOTUs were classified using the SINTAX algorithm (9) with RDPII database as a reference.

# Extraction and characterization of exopolymeric substances (EPS) from microbial mats

EPS samples were lypholized prior to analysis at -50°C and 5 g added to a clean reaction tube. EPS was extracted from lypholized samples by adding up to 15 ml (3:1, solution:sample) of a 10 % (w/v) NaCl solution. The mixture was incubated at 40°C for 15 minutes followed by centrifugation at 4000 rpm for 30 minutes and the supernatant was transferred to a clean reaction tube. This was repeated thrice for each sample and all supernatants were combined. Ice cold ethanol was then added to the supernatant up to a final concentration of 70% and allowed to precipitate at 4°C overnight. After precipitation EPS was pelleted via centrifugation at 4000 rpm for 30 minutes at room temperature twice. Pelleted EPS was purified via dialysis (MWCO 14,000 daltons) against cooled and stirred distilled water for 12 hours at 4 hour intervals where the water was changed. Purified EPS was stored at -20°C prior to analysis (12).

Quality of the extraction and purity of the EPS was evaluated by measuring Adenosine Tripohosphate (ATP) on raw mat samples prior to extraction and on spent mat material following extraction. ATP was extracted from samples using the TIP (0.6 M imidazole in 1.10 M trichloroacetic acid with 0.25 M phosphorous from Na_2_HPO_4_) reagent following the procedure from (10). ATP was measured using Molecular Probes ATP determination kit following the manufacturer’s instructions on a Biotek Synergy 4 microplate reader (USA) on luminescent mode.

Concentrations of carbohydrates, proteins, and uronic acids of EPS were determined using micro-assay colorometric methods measured on a Molecular Devices SpectraMax 384 (USA) microplate reader. Carbohydrates were measured using the phenol-sulfuric acid method (11-13). For this assay EPS extract was mixed with 5% aqueous phenol (w/v) and sulfuric acid (ratio of 2:1:5) and measured spectrophotometrically at 490 nm using glucose as a standard. Proteins were measured using the Bradford assay (14, 13). The assay is performed using solution which consisted of 0.01% (w/v) coomassie brilliant blue g-250, 4.7% (w/v) ethanol, and 8.5% (w/v) phosphoric acid mixed in a 10:1 ratio with sample and measured at 595 nm. Bovine serum albumin (BSA) was used as a standard. Uronic acids were determined using galacturonic acid equivalents (15,13). To perform this assay extracted 6 ml of 0.0125 M Na-tetraborate in 36 N sulfuric acid was added to 1 ml of EPS on ice then heated in a 100°C water bath for 30 minutes. After heating samples were cooled in ice and0.1 ml of 0.15 % (w/v) meta-hydroxydiphenyl in 0.5% (w/v) NaOH, vortexed briefly, and measured after 5 minutes at 520 nm.

Table S1: Geochemical Conditions of KAAS-1 across 3 sampling seasons, 03/16, 10/16and 02/18. Water column was pooled surface water up to 5cm in depth; Sediment water interface (SWI) was the upper 5mm of the mat; Microbial mat was the middle of cored material, usually 4-5cm after the SWI.

| Site | Date  (mm/yy) | Zone and Depth (mm) | T (°C) | Salinity  (‰) | pH | Mg:Ca | Mg  (mmol L^-1^) | Ca  (mmol L^-1^) | | Na  (mmol L^-1^) | K  (mmol L^-1^) | Sr  (mmol L^-1^) | B  (mmol L^-1^) | Si  (mmol L^-1^) |
| --- | --- | --- | --- | --- | --- | --- | --- | --- | --- | --- | --- | --- | --- | --- |
| KAAS-1 | 03/16 | Water Column | Air: 25  Water 25 | 47.41 | 8.2 | 4.71 | 91.36 | 19.38 | 788.42 | | 26.44 | 0.19 | 0.55 | 0.11 |
| KAAS-1 | 03/16 | SWI; 0-0.2 | 25 | 48.13 | 8.8 | 5.35 | 91.45 | 17.09 | 810.46 | | 18.78 | 0.16 | 0.00 | 0.44 |
| KAAS-1 | 03/16 | Microbial Mat | 25 | 48.59 | 6.78 | 5.21 | 91.45 | 17.55 | 854.22 | | 23.15 | 0.16 | 0.38 | 0.44 |
| KAAS-1 | 10/16 | Water Column | Air: 36  Water: 33 | 137.79 | 7.6 | 7.38 | 570.15 | 77.29 | 5088.48 | | 95.66 | 0.01 | 4.11 | BDL |
| KAAS-1 | 10/16 | SWI | 31 | 74.00 | 7.6 | 13.76 | 341.55 | 24.82 | 2727.40 | | 50.11 | 0.03 | 1.39 | BDL |
| KAAS-1 | 10/16 | Microbial Mat | 31 | 72.57 | 7.95 | 15.38 | 331.78 | 21.57 | 2683.08 | | 49.09 | BDL | 1.26 | BDL |
| KAAS-1 | 02/18 | Water Column | Air: 26  Water: 26 | 77.03 | 8 | 9.77 | 265.73 | 27.14 | 1487.83 | | 48.48 | 0.21 | 3.07 | BDL |
| KAAS-1 | 02/18 | SWI | 26 | 39.46 | 8.63 | 3.97 | 196.86 | 49.50 | 1369.01 | | 29.07 | 0.40 | 2.00 | 4.44 |
| KAAS-1 | 02/18 | Microbial Mat | 23 | 25.93 | 6.5 | 11.34 | 112.73 | 9.94 | 957.14 | | 19.34 | 0.14 | 0.82 | 0.02 |

Table S2: Geochemical Conditions of KAAS-2 across 3 sampling seasons, 03/16, 10/16 and 02/18. Water column was pooled surface water up generally <2cm in depth; Sediment water interface (SWI) was the upper 5mm of the mat; Microbial mat was the middle of cored material, usually 4-5cm after the SWI.

| Site | Date  (mm/yy) | Zone | T (°C) | Salinity  (‰) | pH | Mg:Ca | Mg  (mmol L^-1^) | Ca  (mmol L^-1^) | Na  (mmol L^-1^) | K  (mmol L^-1^) | Sr  (mmol L^-1^) | B  (mmol L^-1^) | Si  (mmol L^-1^) |
| --- | --- | --- | --- | --- | --- | --- | --- | --- | --- | --- | --- | --- | --- |
| KAAS-2 | 03/16 | Water Column | Air: 25  Water 25 | 79.45 | 8.16 | 6.20 | 150.99 | 24.34 | 788.42 | 41.27 | 0.29 | 0.87 | 1.21 |
| KAAS-2 | 03/16 | SWI | 25 | 78.63 | 8.5 | 7.18 | 146.97 | 20.46 | 810.46 | 36.30 | 0.26 | 0.75 | 1.34 |
| KAAS-2 | 03/16 | Microbial Mat | 25 | 81.04 | 7.19 | 8.08 | 159.82 | 19.77 | 854.22 | 39.45 | 0.27 | 0.94 | 0.40 |
| KAAS-2 | 10/16 | Water Column | Air: 36  - | - | - | - | - | - | - | - | - | - | - |
| KAAS-2 | 10/16 | SWI | - | - | - | - | - | - | - | - | - | - | - |
| KAAS-2 | 10/16 | Microbial Mat | - | - | - | - | - | - | - | - | - | - | - |
| KAAS-2 | 02/18 | Water Column | Air: 26  Water: 35 | 140.12 | 6.55 | 60.18 | 1869.30 | 31.06 | 7609.47 | 284.40 | 0.05 | 18.56 | 5.01 |
| KAAS-2 | 02/18 | SWI | 34 | 92.30 | 6.70 | 26.50 | 845.58 | 31.90 | 2855.12 | 120.49 | 0.032 | 2.30 | 9.17 |
| KAAS-2 | 02/18 | Microbial Mat | 34 | 102.76 | 6.50 | 123.16 | 1252.63 | 10.17 | 2753.37 | 215.88 | 0.03 | 1.51 | 13.00 |

**
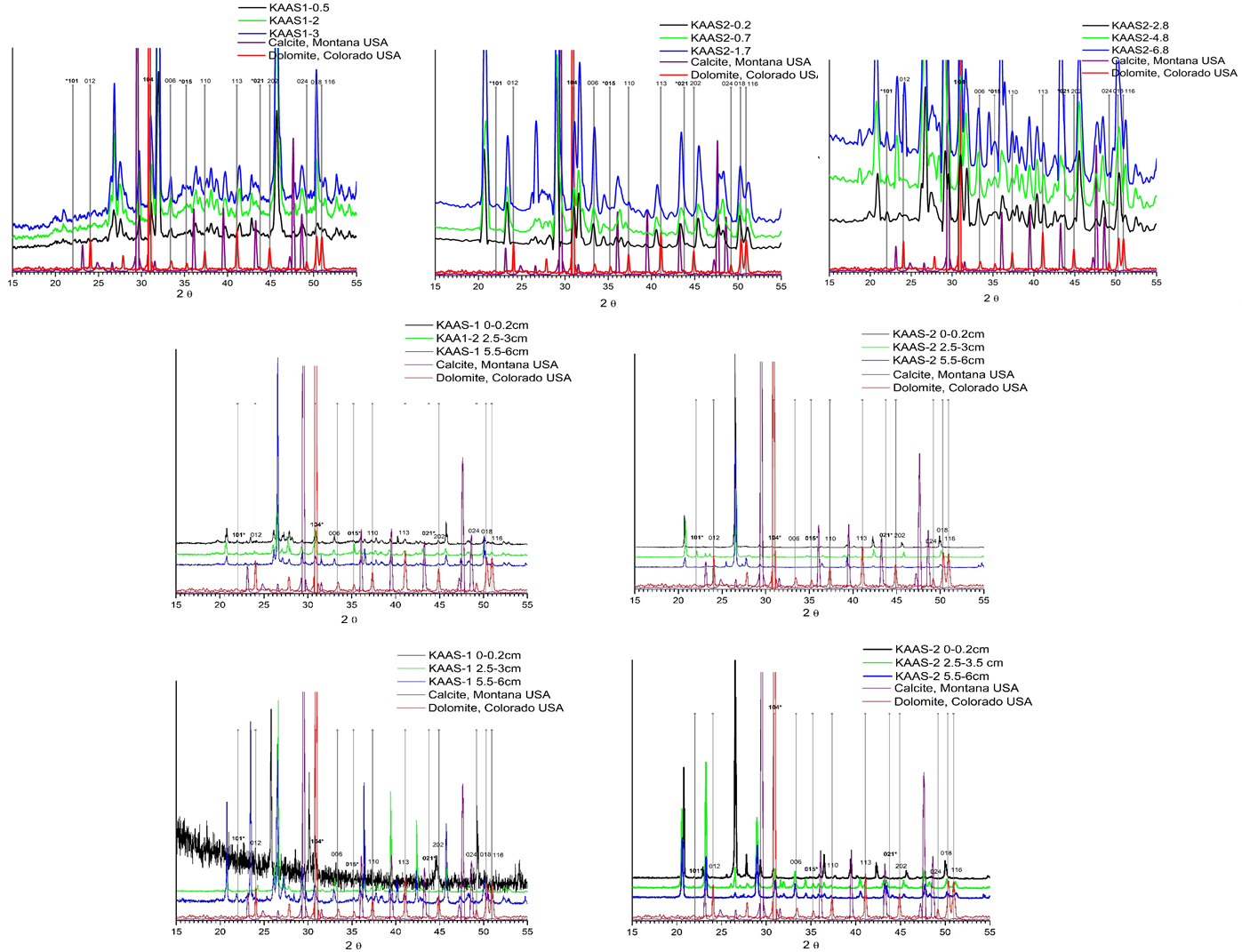
Figure S1:** XRD data across 3 sampling seasons

**Figure S2 (Top):** Colorometric determinations of carbohydrates (black), proteins (red), and uronic acids (blue) from extracted and purified EPS from KAAS-1 and KAAS-2 during March of 2016. **(Middle):** Colorometric determinations of carbohydrates (black), proteins (red), and uronic acids (blue) from extracted and purified EPS from KAAS-1 and KAAS-2 during October of 2016. **(Bottom):** Colorometric determinations of carbohydrates (black), proteins (red), and uronic acids (blue) from extracted and purified EPS from KAAS-1 and KAAS-2 during February of 2018. Samples designated as top represent the green or orange layers from KAAS-1 and KAAS-2 or uppermost 0.5cm, while samples designated as middle represent measurements from the black layer and samples designated as bottom represent measurements taken from the grey layer


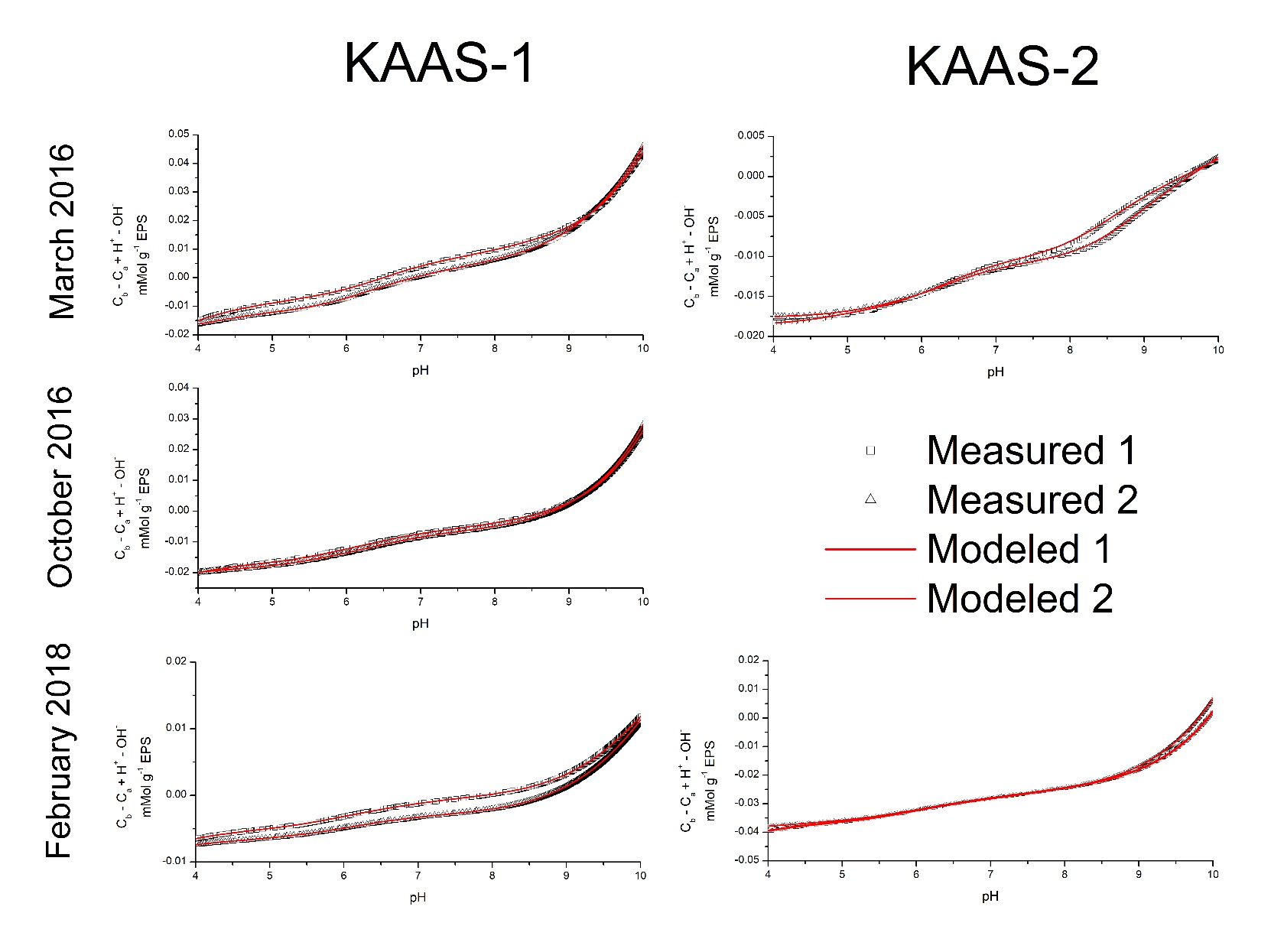


**Figure S3**: Charge excess calculated from titration data across replicates and modeling using LPM from KAAS-1 and KAAS-2 during each of the three sampling seasons.

#
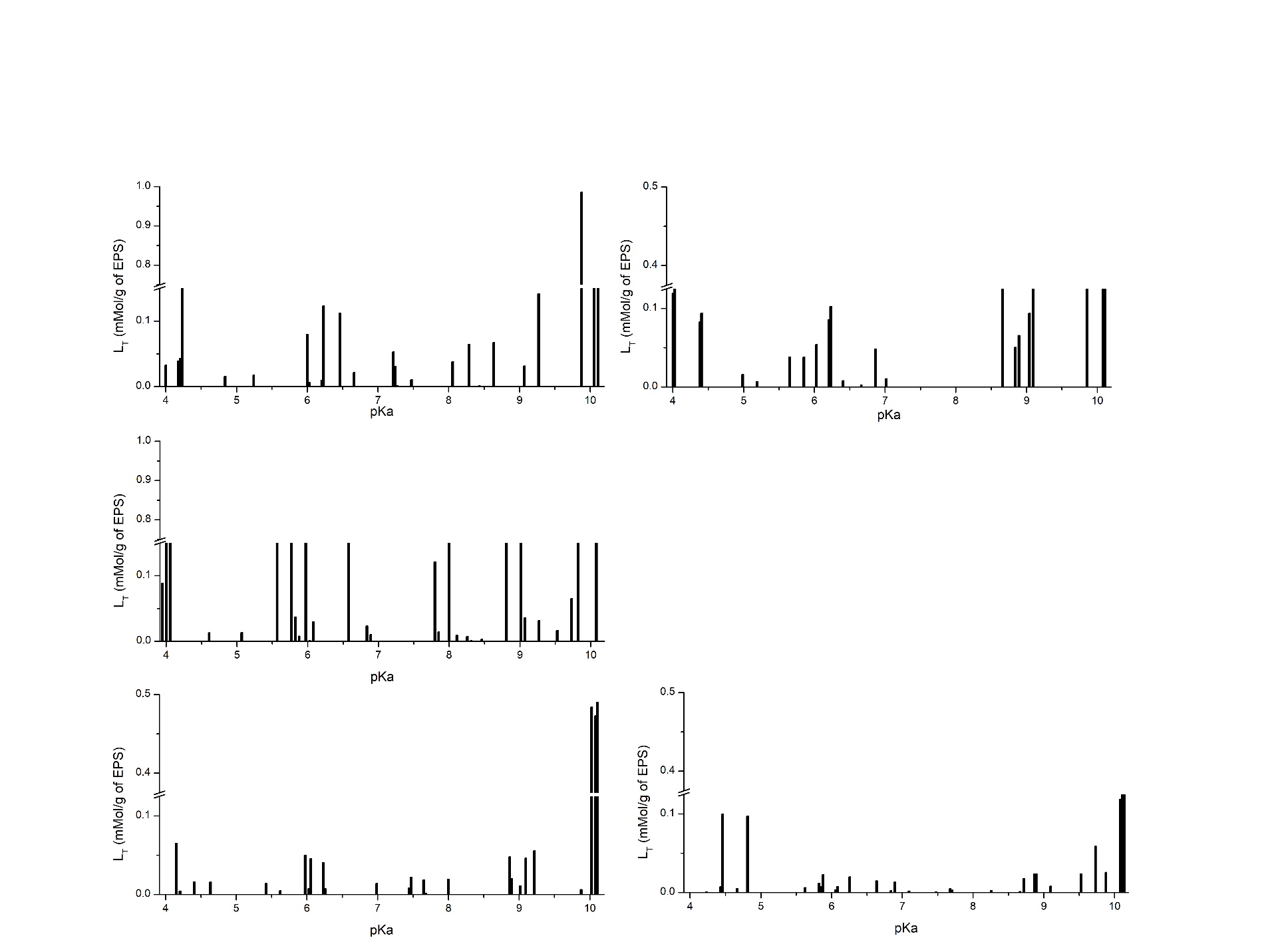


**Figure S4 (Top):** Functional group density and pKa values obtained using LPM of titration data for KAAS-1 and KAAS-2 during March of 2016. **(Middle):** Functional group density and pKa values obtained using LPM of titration data for KAAS-1 and KAAS-2 during October of 2016.  **(Bottom):** Functional group density and pKa values obtained using LPM of titration data for KAAS-1 and KAAS-2 during February of 2018.

# References

1. Z. A. DiLoreto et al., Microbial community composition and dolomite formation in the hypersaline microbial mats of the Khor Al-Adaid sabkhas, Qatar. Extremophiles, 1-18 (2019).
2. J. Sherwood, F. Stagnitti, M. Kokkinn, W. Williams, A standard table for predicting equilibrium dissolved oxygen concentrations in salt lakes dominated by sodium chloride. *International Journal of Salt Lake Research* **1**, 1-6 (1992).
3. V. Salman *et al.*, Calcite-accumulating large sulfur bacteria of the genus Achromatium in Sippewissett Salt Marsh. *The ISME journal* **9**, 2503-2514 (2015).
4. K. Sakai (2015) Routine soil analysis using an Agilent 8800 ICP-QQQ. (Application note 5991-6409EN. Agilent Technologies).
5. J. G. Caporaso *et al.*, QIIME allows analysis of high-throughput community sequencing data. *Nature methods* **7**, 335 (2010).
6. R. C. Edgar, Search and clustering orders of magnitude faster than BLAST. *Bioinformatics* **26**, 2460-2461 (2010).
7. R. C. Edgar, UNOISE2: improved error-correction for Illumina 16S and ITS amplicon sequencing. *BioRxiv*, 081257 (2016).
8. R. C. Edgar, Updating the 97% identity threshold for 16S ribosomal RNA OTUs. *Bioinformatics* **34**, 2371-2375 (2018)..
9. R. Edgar, SINTAX: a simple non-Bayesian taxonomy classifier for 16S and ITS sequences. *BioRxiv*, 074161 (2016).
10. M. Redmile-Gordon, R. White, P. Brookes, Evaluation of substitutes for paraquat in soil microbial ATP determinations using the trichloroacetic acid based reagent of Jenkinson and Oades (1979). *Soil Biology and Biochemistry* **43**, 1098-1100 (2011M. Dubois, K. A. Gilles, J. K. Hamilton, P. t. Rebers, F. Smith, Colorimetric method for determination of sugars and related substances. *Analytical chemistry* **28**, 350-356 (1956).
11. G. Underwood, D. Paterson, R. Parkes, The measurement of microbial carbohydrate exopolymers from intertidal sediments. *Limnology and Oceanography* **40**, 1243-1253 (1995).
12. J.-H. Klock, A. Wieland, R. Seifert, W. Michaelis, Extracellular polymeric substances (EPS) from cyanobacterial mats: characterisation and isolation method optimisation. *Marine Biology* **152**, 1077-1085 (2007).
13. M. M. Bradford, A rapid and sensitive method for the quantitation of microgram quantities of protein utilizing the principle of protein-dye binding. *Analytical biochemistry* **72**, 248-254 (1976).
14. N. Blumenkrantz, G. Asboe-Hansen, New method for quantitative determination of uronic acids. *Analytical biochemistry* **54**, 484-489 (1973).
